# Measuring energy-dependent photoelectron escape in microcrystals

**DOI:** 10.1107/S2052252519016178

**Published:** 2020-01-01

**Authors:** Selina L. S. Storm, Adam D. Crawshaw, Nicholas E. Devenish, Rachel Bolton, David R. Hall, Ivo Tews, Gwyndaf Evans

**Affiliations:** a Diamond Light Source, Harwell Science and Innovation Campus, Didcot OX11 0DE, United Kingdom; b Biological Sciences, Institute for Life Sciences, University of Southampton, Highfield Campus, Southampton SO17 1BJ, United Kingdom

**Keywords:** photoelectron escape, CdTe detector, cryo-TEM sample mounts, microcrystals

## Abstract

With the increasing trend of using microcrystals and intense microbeams at synchrotron X-ray beamlines, radiation damage becomes a more pressing problem. Theoretical calculations show that the photoelectrons that primarily cause damage can escape microcrystals. This effect would become more pronounced with decreasing crystal size as well as at higher energies. To prove this effect, data from cryocooled lysozyme crystals of dimensions 5 × 3 × 3 and 20 × 8 × 8 µm mounted on cryo-transmission electron microscopy (cryo-TEM) grids were collected at 13.5 and 20.1 keV using a PILATUS CdTe 2M detector, which has a similar quantum efficiency at both energies. Accurate absorbed doses were calculated through the direct measurement of individual crystal sizes using scanning electron microscopy after the experiment and characterization of the X-ray microbeam. The crystal lifetime was then quantified based on the *D*_1/2_ metric. In this first systematic study, a longer crystal lifetime for smaller crystals was observed and crystal lifetime increased at higher X-ray energies, supporting the theoretical predictions of photoelectron escape. The use of detector technologies specifically optimized for data collection at energies above 20 keV allows the theoretically predicted photoelectron escape to be quantified and exploited, guiding future beamline-design choices.

## Abstract 2

An increase in crystal lifetime resulting from photoelectron escape was measured on the microfocus beamline I24 at Diamond Light Source for hen-egg white lysozyme microcrystals mounted on cryo-TEM grids at 13.5 and 20.1 keV using a PILATUS CdTe detector.

## 1. Introduction   

With the advances in X-ray focusing techniques and increasing brilliance of the late third and the new fourth generations of synchrotrons, highly intense microbeams have become widely available (Yamamoto *et al.*, 2017 ▸). By matching the beam size to the crystal size in order to optimize the signal-to-noise ratio, data collection from ever smaller crystals becomes possible (Evans *et al.*, 2011 ▸; Holton & Frankel, 2010 ▸), overcoming the requirement for large crystals and enabling new techniques such as time-resolved crystallography (Moffat, 1998 ▸; Šrajer & Schmidt, 2017 ▸) and serial crystallography at synchrotrons (Diederichs & Wang, 2017 ▸; Gati *et al.*, 2014 ▸; Grimes *et al.*, 2018 ▸; Hasegawa *et al.*, 2017 ▸; Heymann *et al.*, 2014 ▸).

Generally, higher flux densities imply that more energy is deposited in the protein crystal, making radiation damage an even more pressing problem. When an X-ray photon is absorbed by a protein crystal, the most likely event is the generation of a photoelectron with a similar energy. This photoelectron is then scattered inelastically within the crystal and produces secondary lower energy electrons, causing further damage (Ravelli & Garman, 2006 ▸). By simulating the paths of photoelectrons, Nave & Hill (2005 ▸) showed that a significant fraction of the high-energy photoelectrons escape the crystal before causing further damage if the crystal size is less than 10 µm and the surrounding material is minimized. The impact of photoelectron escape should be pronounced at higher energies, as photoelectrons are generated with longer path lengths. Example simulations show that increasing the energy from 12 to 25 keV increases the lifetime of a crystal with a linear dimension of 2.5 µm and a 25 Å unit cell by about sevenfold (Cowan & Nave, 2008 ▸).

To exploit the benefits of photoelectron escape, a number of experimental criteria need to be met. Extraneous material surrounding the protein crystal should be minimized, which itself is a source of photoelectrons when illuminated. For crystals mounted in traditional loops the surrounding solvent can scatter a proportion of the escaping photoelectrons back into the crystal. If, however, crystals can be cleanly mounted with minimal solvent then photoelectrons might truly escape into surrounding regions such as air, a conductive mount or in some instances vacuum, not impacting the crystal.

To avoid the transfer of photoelectrons generated in the surrounding material into the crystal and to minimize background scatter, the beam size should match the crystal size. Furthermore, the detector technology must match the setup of the experiment. The vast majority of synchrotron beamlines are nowadays equipped with detectors that use a silicon sensor with a thickness of 450 µm or less. The quantum efficiency of these silicon detectors is reduced substantially at higher energies (Donath *et al.*, 2013 ▸). For example, a 450 µm thick sensor has a quantum efficiency of 98% at 10 keV, of about 61% at 15 keV and of only 33% at 20 keV. Using such a detector at 20 keV nullifies the gains from more photoelectrons leaving the crystal (Nave & Hill, 2005 ▸). However, new hybrid photon-counting detectors using CdTe as a sensor material can be used as they provide quantum efficiencies of higher than 95% in the range 10–30 keV (Zambon *et al.*, 2018 ▸). Another positive effect at higher energies is an increase in the percentage of elastically scattered photons contributing to the diffraction spots (Dickerson & Garman, 2019 ▸; Fourme *et al.*, 2012 ▸; Shimizu *et al.*, 2007 ▸). This increase in diffraction efficiency, which is 76% between 12.4 and 35 keV for a protein crystal with no elements heavier than sulfur, can be exploited using these types of detectors, as shown in recent simulations (Dickerson & Garman, 2019 ▸).

Previous attempts to investigate photoelectron escape experimentally focused on the penetration depth and the spatial distribution of photoelectron damage in large crystals with a range of different beam sizes and shapes (Finfrock *et al.*, 2013 ▸; Sanishvili *et al.*, 2011 ▸). Here, we present the first systematic measurement of photoelectron escape in microcrystals. The results illustrate the benefits of collecting data from microcrystals at higher energies with a suitable detector.

## 2. Materials and methods   

To minimize the material surrounding the protein crystals, cryo-TEM grids were used as crystal carriers. By using these mounts, the crystal sizes can be measured in a scanning electron microscope (SEM) after data collection, enabling an accurate calculation of the dose. The use of a PILATUS CdTe 2M detector exploits the nearly constant quantum efficiency at the used energies of 13.5 and 20.1 keV. Crystal lifetimes are commonly defined by the dose that is required to halve the diffracting power (Owen *et al.*, 2006 ▸). In order to quantify the effects of photoelectron escape the *D*_1/2_ metric described in Section 2.6 is used.

### 2.1. Sample preparation and sample mounting   

Lysozyme microcrystals were produced using a batch crystallization method (adapted from Martin-Garcia *et al.*, 2017 ▸) in which the size of the microcrystals was controlled by both protein concentration and temperature. Lyophilized hen egg-white lysozyme was purchased from Sigma–Aldrich and was dissolved in 20 m*M* sodium acetate pH 4.6. Crystallization was carried out at 24°C at protein concentrations of 20 mg ml^−1^ to grow larger microcrystals and 50 mg ml^−1^ to grow smaller microcrystals. Both the precipitant solution, which consisted of 1 *M* sodium acetate pH 3.5, 6% PEG 6000, 20% sodium chloride, and the protein solution were incubated at 24°C for 15 min in 1.5 ml tubes. Precipitant and protein solutions were then mixed in a 4:1 ratio and immediately vortexed for 10 s before incubation for 1 h at room temperature. The resulting crystals were approximately 20 × 8 × 8 µm in size for the larger crystals and 5 × 3 × 3 µm for the smaller crystals.

To achieve an appropriate density of microcrystals with isolated single crystals, the stocks were diluted using a solution consisting of 20 m*M* sodium acetate pH 4.6 (20%) and precipitant solution (80%). A starting dilution of 10% of the stock of microcrystals was used and the grids were assessed after plunge-freezing. Further dilution was performed until isolated single crystals could be observed; this was sometimes 1% of the stock of microcrystals. The microcrystals were mounted onto the supporting carbon film of 200 mesh copper Quantifoil R2/2 transmission electron microscope (TEM) grids using a Leica GP2 plunge freezer. Within the sample chamber at 90% humidity, 2 µl of dilution buffer was applied to the back (copper) side of the grid before 2 µl of crystal slurry was applied to the carbon side; blotting for approximately 6 s preceded immediate flash-cooling into liquid ethane. The grids were transferred into liquid nitrogen for storage.

An example of a grid from each batch was imaged using a Jeol JSM-IT100 SEM fitted with a Quorum PP3000T cryo-stage/transfer system to confirm the quality of the blotting by assessing residual liquid, crystalline ice contamination and the crystal-loading density and to confirm the dimensions of the crystals in the batch. Grids were imaged using the same method post X-ray exposure to determine the crystal sizes of the irradiated samples.

### 2.2. Beamline setup and beam characteristics   

The data presented here were collected in three sessions on beamline I24 at Diamond Light Source. To characterize the beam, the photon flux was measured each time using a 500 µm silicon PIN diode (Owen *et al.*, 2009 ▸). The beam sizes used in data collection were determined by performing a knife-edge scan on a gold wire followed by analytical determination of the full width at half maximum (FWHM) of the Gaussian-shaped beam (see Table 1 ▸ and Supplementary Fig. S1 for an example plot). Owing to the setup of I24, the flux does not change when the beam size is changed (Evans *et al.*, 2011 ▸).

### 2.3. Detector setup   

A pixel-array detector equipped with a 1 mm thick CdTe sensor (PILATUS 3X 2M CdTe, Dectris) was used, exploiting the similar quantum efficiency at the energies of interest. Currently, this detector is calibrated for the use of energies higher than 16 keV and allows a maximum count rate of 1 × 10^7^ photons s^−1^ in each pixel. To use the detector at 13.5 keV, the energy threshold was set manually to 6.75 keV and the flat-field correction was switched off. In order to obtain a better flat field than the extrapolated one normally applied internally within the detector, a flat field was calculated based on data collected from a germanium-doped glassy sample. Ideally, this flat field would have been recorded from a strontium or rubidium sample providing X-ray emission lines close to 13.5 keV. Attempts to perform this using a strontium pellet sealed in Kapton failed owing to artefacts arising from the supporting material. Nevertheless, the histogram of the pixel counts versus the number of pixels shows a tighter distribution, indicating that the newly recorded flat field from the germanium-doped glassy sample performs better than the extrapolated flat field.

### 2.4. Data collection   

**Figure 1:**
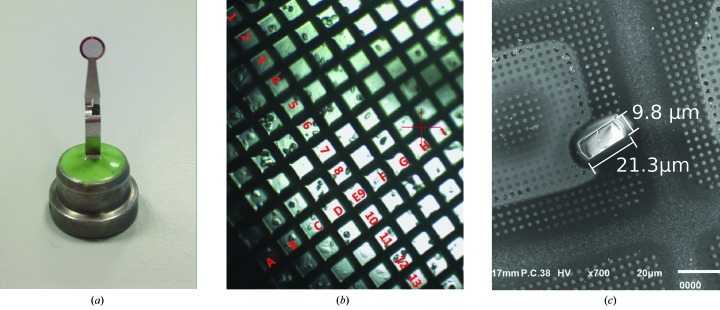
(*a*) A cryo-TEM grid held on a tweezer pin. (*b*) A cryo-TEM grid viewed in the on-axis viewing microscope overlaid with the coordinate system used to identify crystals in the scanning electron microscope (SEM) post X-ray exposure. (*c*) SEM image of HEWL crystal dimensions being determined.

The cryo-TEM grids were mounted on tweezer pins on the goniometer [Fig. 1 ▸(*a*)]. Centring of the smaller crystals proved to be difficult owing to the limited resolution of the on-axis viewing (OAV) microscope. To measure the crystal sizes in the SEM after data collection, a coordinate system was generated to identify the crystals [Fig. 1 ▸(*b*)]. Identical sweeps of 5° each (0.1° per frame) were recorded at 100 K, ensuring an even illumination of the crystals during rotation. These sweeps were repeated 8–25 times to determine a reliable decay curve. The cryo-TEM grids were recovered after data collection and were stored in liquid nitrogen. Data at 20.1 keV were always measured with 100% transmission, with exposure times ranging from 0.05 to 0.2 s per frame. At 13.5 keV, the transmission was varied in combination with exposure times ranging from 0.01 to 0.02 s per frame to exclude effects arising from the dead time of the detector, ensuring the collection of comparable doses per sweep. The detector distance was set to allow measurements to a resolution of 1.8 Å at both energies. As an example, the first image of a data set from a small crystal collected at both energies can be found in Supplementary Fig. S2.

### 2.5. Data analysis   

The data were processed with the *DIALS* v.1.4 integration package (Winter *et al.*, 2018 ▸). The flat-field correction was applied manually to the raw image data collected at 13.5 keV using a Python script. Powder diffraction rings arising from the copper cryo-TEM grids were removed using masks at resolutions between 2.05 and 2.10 Å and between 1.77 and 1.81 Å to prevent bias in the integrated intensity counts. For sessions A and C data were measured at several different detector positions, and the experimental geometry was determined by joint refinement of the first sweep of data from each crystal at that distance; for session C the beam centre was fine-tuned by manual alignment to the powder rings. For session B, two reference 180° cubic insulin data sets were measured to assist with the accurate determination of the detector geometry and the beam centre. Once the correct geometry had been determined, indexing was attempted on the first sweep for each lysozyme crystal using the known crystal unit cell and space group. Diffraction data that did not index with this information were discarded, as were data that showed evidence of multiple lattices. After indexing, the experimental geometry was refined and the data were integrated. For subsequent sweeps of data from the same crystal, diffraction spots were indexed with fixed geometry using the known crystal unit-cell and orientation parameters refined from the previous sweep. This approach ensured the stability of lattice determination and orientation, especially as the diffraction spots became progressively weaker with increasing sweep number. The crystal and experimental geometry for each sweep were then re-refined and integrated, and the updated geometry model was used for the subsequent sweep. This process was repeated until the diffraction spots became too weak to identify and indexing failed. To ensure comparability, data were discarded for crystals where profile-fitting of the Bragg reflections failed. After integration the data were scaled using *dials.scale* in order to estimate the resolution, as defined by CC_1/2_ > 0.33. This resolution was then used as a cutoff to calculate the total integrated unscaled intensities and variance for each crystal sweep. Representative data-collection and data-processing statistics for each of the three sessions can be found in Supplementary Table S1.

### 2.6. Determination of the dose and *D*_1/2_   

For those grids that were successfully recovered (9 out of 12) the crystal sizes were measured in the SEM. Based on the morphology of the crystals, it was assumed that the third dimension of the crystals perpendicular to the grid surface was equal to their smallest dimension parallel to the surface. For those cases where the grid was not recovered the crystal size was estimated to be the average crystal size of all measured crystals from the same batch. In a few cases the crystals were completely destroyed by radiation damage (compare Supplementary Fig. S3) and the average crystal size was again used to estimate these crystal sizes. The average diffraction-weighted dose, from now on referred to simply as the dose, was calculated with *RADDOSE*-3*D* (Zeldin *et al.*, 2013 ▸), not taking photoelectron escape (Bury *et al.*, 2018 ▸) into account unless explicitly stated otherwise. Assuming an estimated error of 2^1/2^% for the flux based on 1% measurement inaccuracy and 1% inaccuracy of the diode, an error of 0.1 µm in the measured crystal dimensions and an error of 0.5 µm for the third dimension of the small crystals, the error in the dose per sweep ranges between 1.5% and 3.6%, assuming a constant average photon flux and a constant beam size.

**Figure 2:**
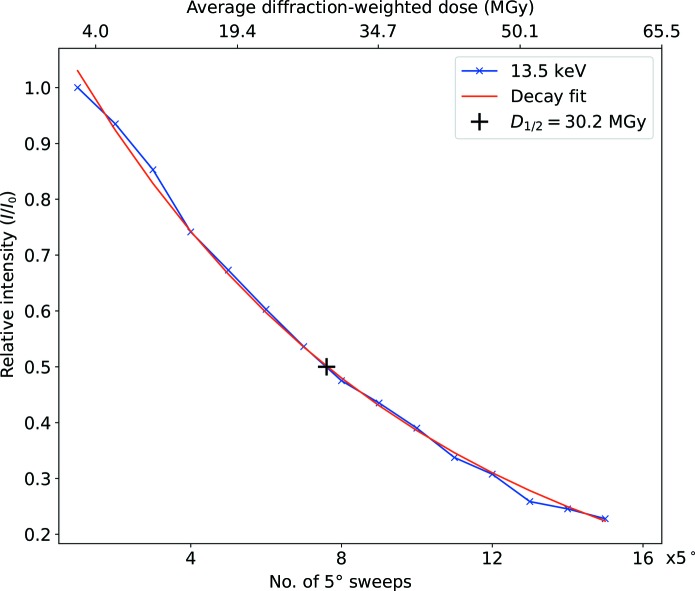
Determination of *D*_1/2_ based on the fitted decay curve of the normalized intensities.

To determine the dose where the total integrated intensity of a 5° sweep of data decreases to half of its initial value (*D*_1/2_), the data were normalized to the total integrated intensity of the first 5° of data (*I*_0_) and then fitted with an exponential decay curve of the type
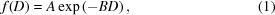
where *D* is the average diffraction-weighted dose per 5° sweep (Fig. 2 ▸). The *D*_1/2_ value was determined from the fit and the errors of the fitting parameters were determined using the least-squares method. Table 2 ▸ gives an overview of the numbers of crystals used.

## 3. Results   

### 3.1. Measuring photoelectron escape   

**Figure 3:**
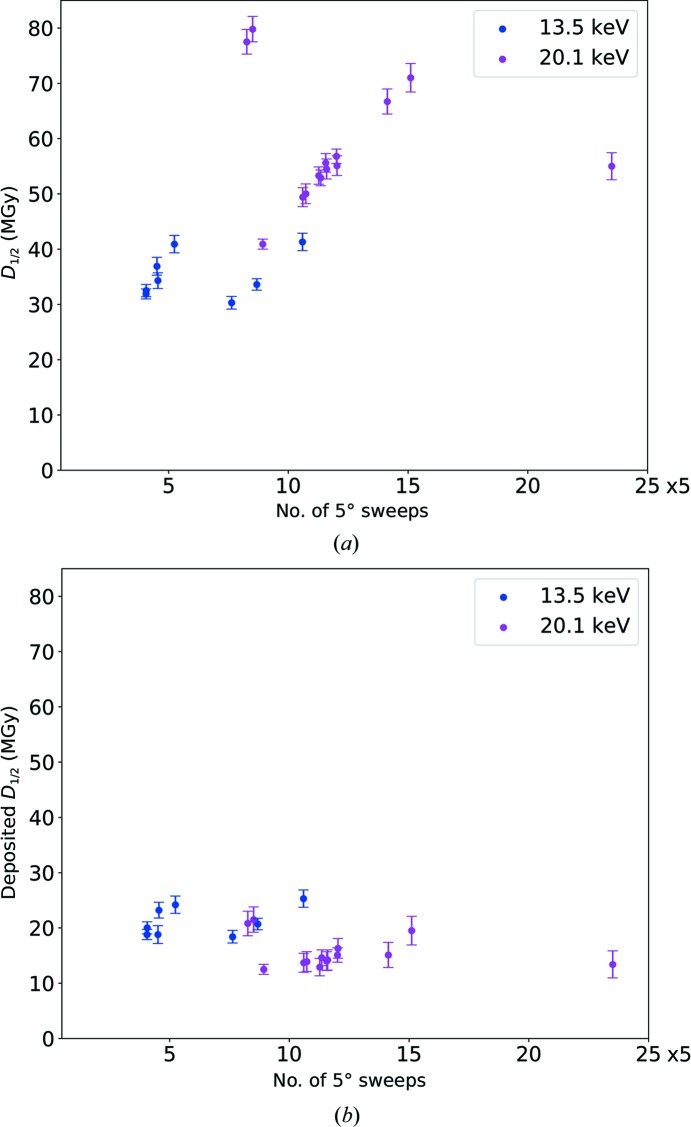
(*a*) *D*_1/2_ values for 22 crystals with an average size of 5.2 × 3.1 × 3.1 µm with a beam size of 6.4 × 7.2 µm collected from the same grid at 13.5 keV (blue) and at 20.1 keV (magenta) plotted with error bars representing measurement errors in flux, crystal size and fit of the decay curve. (*b*) As in (*a*) but plotted against the dose corrected for photoelectron escape, referred to as the deposited dose.

Crystal diffraction typically extended to a resolution limit (judged by the CC_1/2_ > 0.33 criterion) of between 1.6 and 2.2 Å, with the exception of a few outliers. The estimation of the resolution limit by *DIALS* takes data in the corner of the detector into account. It was possible to successfully obtain *D*_1/2_ values for 106 crystals from 12 grids out of a total of 202 measured crystals. The relatively large proportion of outlier data sets are owing to issues related to both inconsistencies in sample preparation and the effects of storage-ring current top-up occasionally taking place during data collection. The effect of high X-ray background for data sets measured on ‘wetter’ grids made data analysis more challenging, especially for the small crystal data sets, and full decay series were often discarded because of this [compare Supplementary Fig. S4(*a*)]. The storage-ring current top-up of 20 ± 2 s leads to a varying beam size and with that to a varying illumination of the crystals, which contributed to noisy decay curves and unreliable dose measurements (compare with Supplementary Fig. S4*b*). Furthermore, data analysis occasionally resulted in an apparent high mosaicity [as indicated by refined r.m.s.d.(*z*) values of >1°] that either reflected poor intrinsic crystal quality for these cases or a low signal-to-noise ratio in the data. All decay series that did not reach half intensity, had r.m.s.d.(*z*) > 1 or displayed clear signs of corruption from topup were discarded. For illustration, the following analysis describes a grid where it was possible to collect many data sets from small crystals, eight at 13.5 keV and 14 at 20.1 keV, removing any systematic effects that may have been introduced by grid-to-grid crystal variation. Despite the spread of *D*_1/2_ values, Fig. 3 ▸(*a*) shows a clear separation of data points representing the 13.5 and 20.1 keV data sets into two clusters. The *D*_1/2_ values for the data collected at 20.1 keV from this grid average 58.5 MGy, while the average *D*_1/2_ value at 13.5 keV is 35.2 MGy. The average *D*_1/2_ value obtained from the data collected at 20.1 keV is thus 66% higher than that from the data collected at 13.5 keV. The same data corrected for photoelectron escape with *RADDOSE*3*D* are shown in Fig. 3 ▸(*b*). Referring to this corrected dose as the deposited dose, it can be seen that the resulting deposited dose *D*_1/2_ values are similar at both X-ray energies. The average deposited dose *D*_1/2_ values are 21.2 ± 2.7 MGy at 13.5 keV and 15.5 ± 2.9 MGy at 20.1 keV. Taking a factor of two between *RADDOSE* v.2 and *RADDOSE*-3*D* into account, these values are close to the half dose of 43 MGy reported by Owen *et al.* (2006 ▸) that used *RADDOSE* v.2.

**Figure 4:**
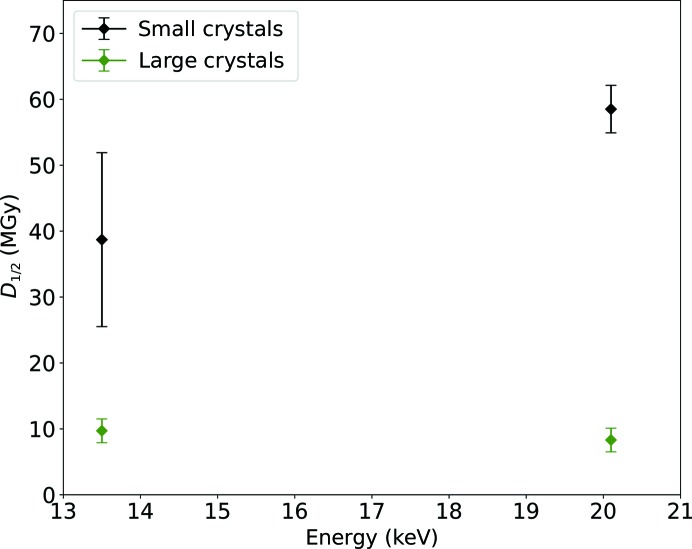
Average *D*_1/2_ values from all 106 crystals plotted for both crystal sizes and energies. The error bars correspond to the standard deviations of the *D*_1/2_ values.

Fig. 4 ▸ shows the final analysis, giving the average *D*_1/2_ values and their standard deviations for the two different crystal sizes for both energies from all 106 crystals. The *D*_1/2_ values from the other grids were more self-consistent than that chosen for illustration, as can be seen from the standard deviation relative to the average *D*_1/2_ value, which ranged between 10.8% and 29.4%. The large standard deviation for the small crystals collected at 13.5 keV is owing to the fact that the data were collected from three different grids, one of which showed much lower *D*_1/2_ values (the reasons for this are discussed below). Still, the data presented here for small crystals show a *D*_1/2_ increase of 51.0 ± 39.2% at 20.1 keV compared with 13.5 keV.

Additionally, the average *D*_1/2_ values of the data collected at 13.5 keV differ by a factor of 4.7 between the small and the large crystals and by a factor of six relative to the data collected at 20.1 keV. The *D*_1/2_ values from the larger crystals are more consistent owing to the better signal-to-noise-ratio, and they do not show significant variation with energy. Based on Monte Carlo simulations of photoelectron escape (Dickerson & Garman, 2019 ▸; Cowan & Nave, 2008 ▸), the difference between the *D*_1/2_ values for the small crystals should be in the range 40–50%, which is similar to that observed here.

### 3.2. Error calculation and reasons for variations in *D*_1/2_   

To quantify the problem of the noisiness of some decay curves, the errors in the fitting parameters *A* and *B* in (1) were calculated and included in the error calculation. The errors in *D*_1/2_ calculated by error propagation for the grid with the most small crystals on it range between 2.2% and 4.5% (compare Fig. 3 ▸). A relationship between the slightly different beam sizes used during the different sessions and *D*_1/2_ could not be found. The reason for a systematic difference in *D*_1/2_ between grids is most likely to be related to the handling of the grids. Owing to the difficulties in handling them reproducibly, the grids were subjected to variable humidity and temperature at the beamline, both of which can affect the quality of lysozyme microcrystals (Kriminski *et al.*, 2002 ▸).

To further understand the spread in the *D*_1/2_ values, the resolution of the data, the thickness of the solvent around the crystals and the alignment of the grid in the cryostream were considered. The crystals depicted in Fig. 3 ▸ all diffracted to 1.7–2.2 Å resolution and the difference in resolution was investigated by the calculation of normalized *D*_1/2_ values as described by Leal *et al.* (2013 ▸). These normalized *D*_1/2_ values show approximately the same spread for this resolution range, which leads to the conclusion that the accuracy of the *D*_1/2_ values cannot be improved using this model, which was developed for room-temperature data sets. In addition, the thickness of the surrounding solvent layer of 15 small crystals of very similar size but deviating *D*_1/2_ values was probed by measuring it in the SEM, resulting in average water thicknesses ranging from 6.5 to 11 µm. An obvious correlation between the water thickness and the *D*_1/2_ values could not be found, which is in agreement with the values obtained from *RADDOSE*-3*D* v.4.0, indicating a maximum difference of 2.5% per sweep at 20.1 keV and 0.2% at 13.5 keV. In summary, the variations within the *D*_1/2_ values of the small crystals from one grid are best explained by crystal-to-crystal variation and crystal alignment.

With a width of 3 mm, cryo-TEM grids are also challenging to align in the cryostream. If the grid is very slightly angled, the apparent diameter of the cryostream is reduced, the crystals could heat faster (Warren *et al.*, 2019 ▸) and the lifetime of the crystals would be systematically reduced. This might have been the case for one of the grids with small crystals collected at 13.5 keV.

In summary, the variations in the *D*_1/2_ values of the small crystals from one grid are best explained by crystal-to-crystal variation and problems with aligning the crystal and the beam. Owing to the limited number of data frames from each crystal, a more thorough investigation of the usual data-quality parameters is not possible.

## 4. Discussion and outlook   

This is the first systematic study showing the effect of photoelectron escape in microcrystals. The study illustrates significant effects arising simply from the handling and mounting of microcrystals. The cryo-TEM grids used with the supporting carbon film provide a sample carrier that allows the mounting of samples on the supporting carbon film with very little surrounding material, but the handling of these grids at a standard beamline is difficult and not very reproducible, being sensitive to temperature and humidity changes. With a width of 3 mm, cryo-TEM grids are challenging to align in the cryostream, and smaller sample holders might be beneficial.

Controlling the amount of surrounding liquid is a further challenge. Novel sample-loading methods and mounts might therefore be explored, such as acoustic dispersion (Davy *et al.*, 2019 ▸). Alternatively, beamlines such as VMXm at Diamond Light Source, for which the mounting methods described here have been developed, have an SEM integrated into the evacuated sample environment and are ideal for such experiments.

An attempt was was made to calculate the doses as accurately as possible and to consider the potential sources of error. However, these calculations assume that the beam size and photon flux are constant. To verify this assumption, both the flux and beam size would have to be monitored at all times, which was not possible at the time of the experiment.

The small difference between the experimental results of our study and simulations (Dickerson & Garman, 2019 ▸; Cowan & Nave, 2008 ▸) could be owing to systematic errors that are not fully accounted for in this analysis or to limitations of the simulations. Additional studies will be required to fully understand these minor discrepancies.

Further questions regarding photoelectron escape remain. The metric used here only quantifies global radiation damage and is based on small sweeps of data. The effect of photoelectron escape on specific radiation damage in microcrystals, such as the reduction of metals and the breakage of disulfide bonds, still remains to be investigated.

A PILATUS 2M CdTe detector enables better exploitation of the benefits of photoelectron escape at high energies. The reduced quantum efficiency of silicon-based detectors hampers the reliable counting of photons, which is crucial when using microbeams and microcrystals. From a crystallo­grapher’s perspective, it would be desirable if the new CdTe detectors were also calibrated to lower energies and covered energies down to the selenium edge, which is still used regularly to introduce sufficient anomalous signal to solve *de novo* structures.

Despite the errors arising from operating the beamline at its current limits, this study provides long-awaited evidence for the photoelectron-escape effect in microcrystals and illustrates the benefits of collecting data at ∼20 keV. These results are worth considering in the design of new microfocus beamlines.

## Supplementary Material

The supporting information shows beam profiles, diffraction images, a representative table of data-collection parameters and data-processing statistics as well as an SEM image of a crystal destroyed by radiation damage. DOI: 10.1107/S2052252519016178/lz5031sup1.pdf

## Figures and Tables

**Table 1:** Beam sizes (FWHM) of the Gaussian-shaped beam and the fluxes measured with a 500 µm thick silicon PIN diode for the three different sessions reported here

Beamtime session	Small beam (µm)	Large beam (µm)	Flux at 13.5 keV (photons s^−1^)	Flux at 20.1 keV (photons s^−1^)
A	9.1 × 8.2	21.9 × 18.2	2.2 × 10^12^	4.2 × 10^11^
B	7.2 × 6.4	n.a.	1.8 × 10^12^	4.9 × 10^11^
C	n.a.	23.4 × 20.5	2.3 × 10^12^	5.5 × 10^11^

**Table 2:** Number of crystals for which the *D*_1/2_ value could be determined Small crystals refer to an average crystal size of 5 × 3 × 3 µm and large crystals to an average crystal size of 20 × 8 × 8 µm.

Energy (keV)	Small crystals	Large crystals	
13.5	23	35	
20.1	14	34	
