# Supplementary material for: Measuring energy-dependent photoelectron escape in microcrystals
Source: IUCrJ. 2020 Jan 1;7(Pt 1):129–35. doi: 10.1107/S2052252519016178 (PMC6949606; doi:10.1107/S2052252519016178)

IUCrJ

Volume 7 (2020)

Supporting information for article:

Measuring energy-dependent photoelectron escape in microcrystals

Selina L. S. Storm, Adam D. Crawshaw, Nicholas E. Devenish, Rachel Bolton, David R. Hall, Ivo Tews and Gwyndaf Evans

Supplementary material

Fig. S1: Gaussian beamprofile for the a) horizontal beamsize of 7.2 µm and b) the vertical beamsize of 6.4 µm for beamtime B obtained by performing a scan on a gold wire with 1 µm resolution and an illumination of 1 s per data point.

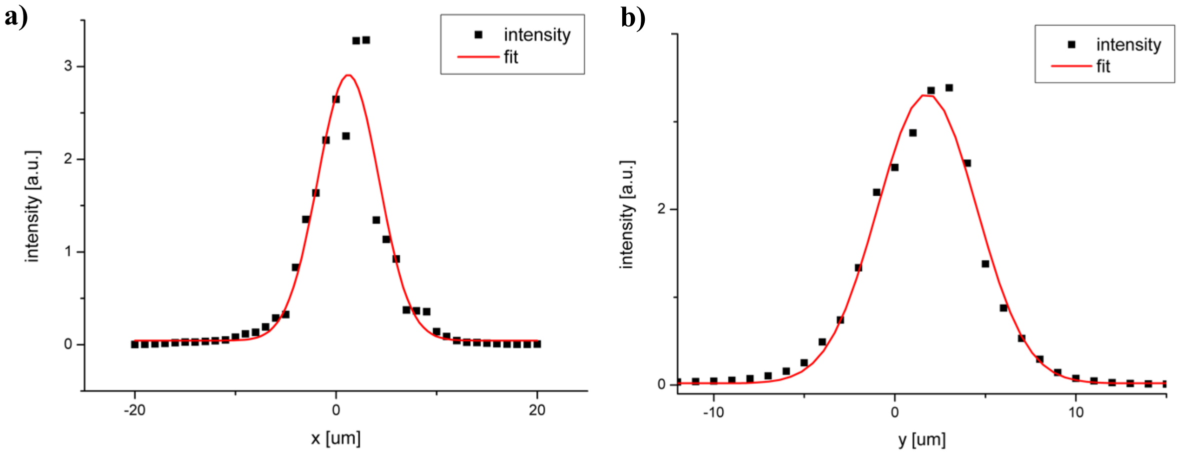

Fig. S2: a) First diffraction image from a lysozyme crystal with a size of 4.6 x 3.5 x 3.5 µm^3 collected at 13.5 keV; b) first diffraction image from a lysozyme crystal with a size of 5.1 x 3.2 x 3.2 µm^3 collected at 20.1 keV.

a)

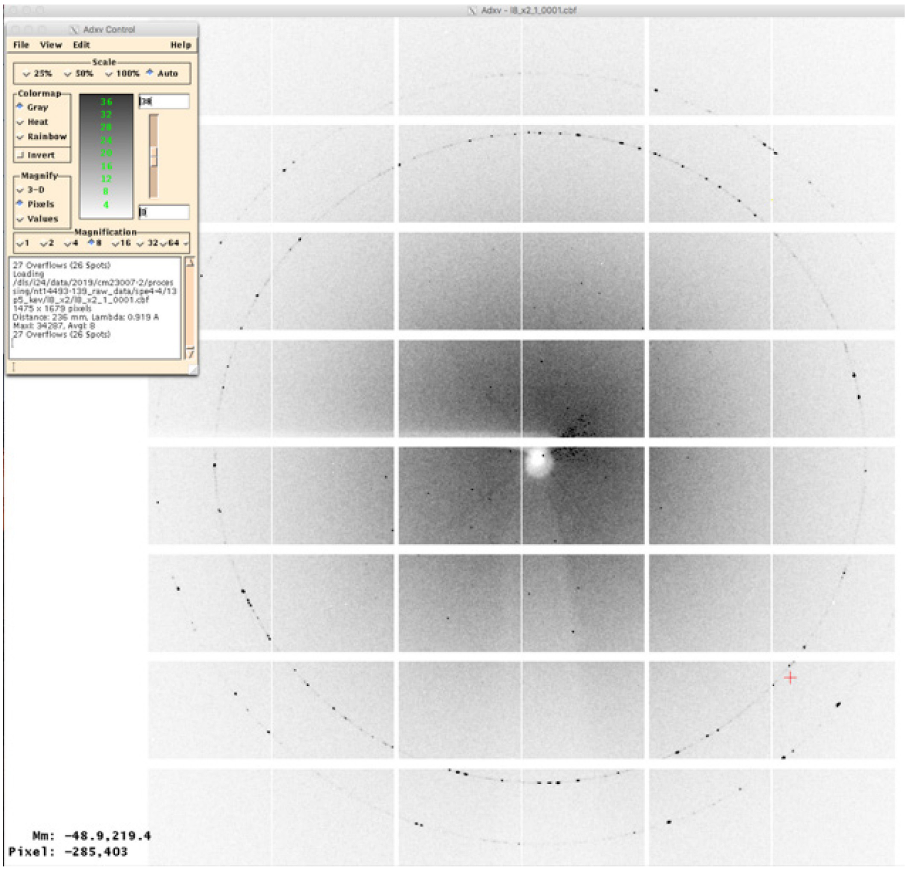

b)

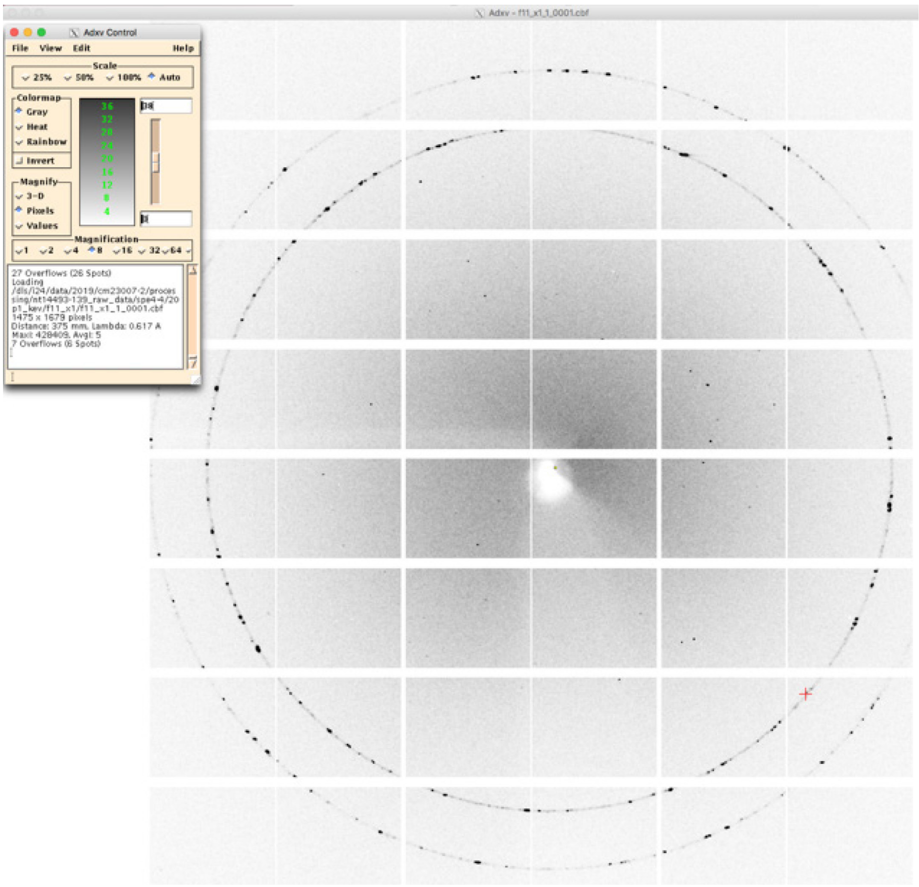

Table S1 a) Representative data collection parameters for the different beamtimes. The flux is the actually applied flux, i.e. the applied transmission is taken into consideration. A sweep refers to 5 degrees of data. The doses are average diffraction weighted doses; the deposited dose takes photoelectron escape into account.

	crystal size [µm^3]	energy [keV]	flux* [ph/s]	exposure time / frame [s]	beam size [µm^2]	Dose/ sweep [MGy]	no of sweeps to $D_{1/2}$	$D_{1/2}$ [MG]	deposited $D_{1/2}$ [MGy]
Beamtime A	5.4 x 3.2 x 3.2	13.5	4.5x10^{11}	0.05	9.1 × 8.2	4	13.4	54.1	33
	20.8 x 7.8 x 7.8	20.1	4.2x 10^{11}	0.15	21.9 x 18.2	0.7	19	12.4	7.9
Beamtime B	5.4 x 3.3 x 3.3	13.5	9.1x10^{11}	0.02	7.2 x 6.4	3.9	10.6	41.3	25.3
	5.1 x 2.8 x 2.8	20.1	4.9x10^{11}	0.1	7.2 x 6.4	4.7	11.6	54.5	14.2
Beamtime C	22.9 x 8.6 x 8.6	13.5	1.7x10^{12}	0.02	23.4 x 20.5	0.7	7.8	5.6	4.8
	23 x 9.3 x 9.3	20.1	5.5 x 10^{11}	0.16	23.4 x 20.5	0.8	7.4	5.7	4

Table S1 b) Processing statistics are taken from the scaled 5 degree sweeps by applying a resolution cut-off at 3 Å for comparability. The number in brackets refers to the highest resolution shell in the range of 0.05 Å. The sweep reaching $D_{1/2}$ is defined as the rounded up value given in table 1a. The resolution cut-off is based on the $CC_{1/2}$ >= 0.33.

	first sweep					sweep reaching $D_{1/2}$			
	resolution [Å]	I/σ	R_{meas}	$CC_{1/2}$	mosaicity [°]	resolution [Å]	I/σ	R_{meas} [%]	$CC_{1/2}$ [%]
Beamtime A	2.0	5.0 [1.1]	0.17 [1.4]	0.96 [0.0]	0.12	2.9	2.9 [0.4]	0.27 [1.37]	0.90 [-0.98]
	2.2	37.0 [15.5]	0.14 [0.19]	0.98 [0.92]	0.11	2.2	23.1 [6.3]	0.22 [0.15]	0.97 [0.90]
Beamtime B	1.9	4.7 [1.8]	0.14 [0.11]	0.97 [0.95]	0.11	2.5	2.6 [0.4]	0.26 [0.56]	0.85 [0.84]
	1.9	6.2 [2.4]	0.09 [0.22]	0.98 [0.90]	0.09	2.8	2.3 [0.3]	0.27 [0.95]	0.74 [-0.18]
Beamtime C	1.9	35.2 [15]	0.09 [0.11]	0.97 [0.98]	0.11	2.0	26.4 [8.5]	0.07 [0.14]	0.98 [0.9]
	1.9	29.5 [11.7]	0.16 [0.10]	0.90 [0.99]	0.24	2.0	24.1 [8.7]	0.18 [0.11]	0.86 [1.0]

Fig. S3: SEM image of a destroyed crystal. For comparison, three intact crystals in the upper half of the picture.

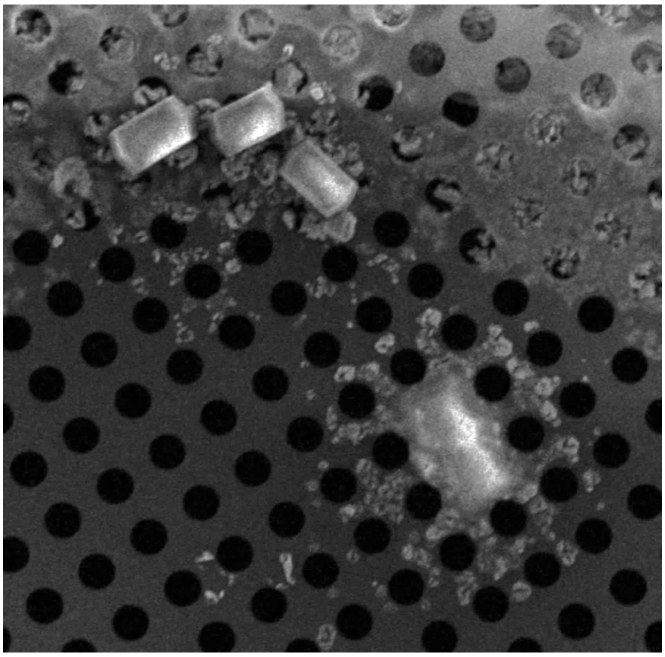

Fig. S4 a: The plot shows the total integrated intensity of all profile-fitted reflections in total (blue) and for the different resolution shells. The gaps indicate that the integration of the profile-fitted reflection was not possible. Obviously, these data could not be used in the final analysis.

a)

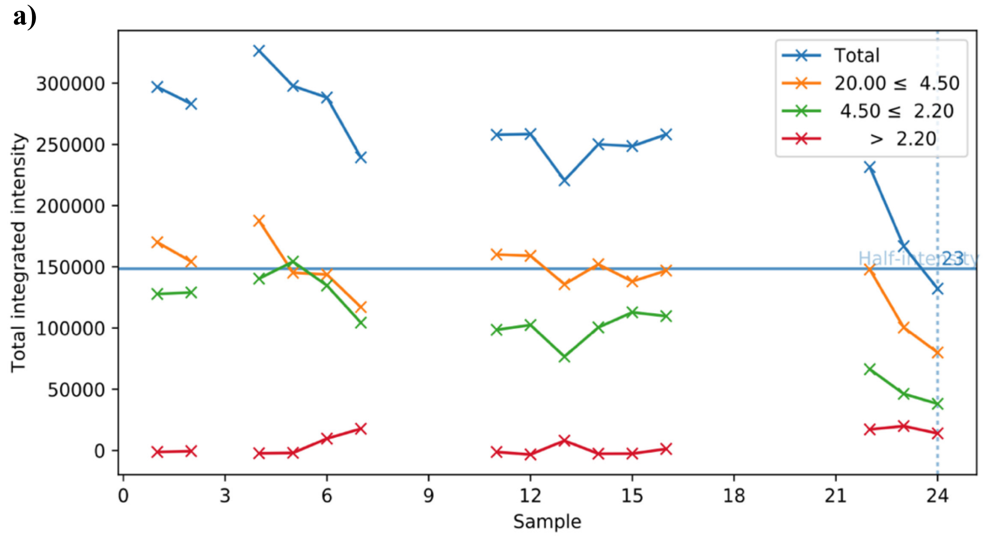

Fig. S4 b: As in Fig. S4 a, but collected during the injection of electrons into the ring (top-up). This leads to an instable and enlarged beam, which is why neither the doses could be determined correctly nor a reasonable decay plot could be obtained.

b)

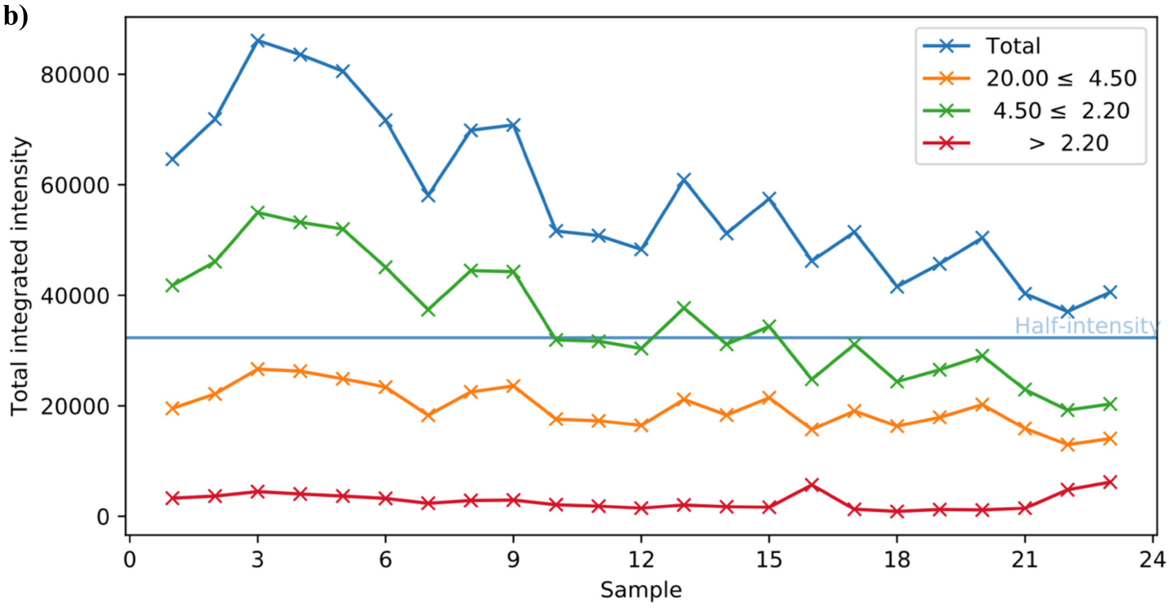

Supplement: Supplementary file 1 [file m-07-00129-sup1.pdf]
